# Supplementary material for: Polarization conversion in bottom-up grown quasi-1D fibrous red phosphorus flakes
Source: Nat Commun. 2023 Jul 20;14:4398. doi: 10.1038/s41467-023-40122-2 (PMC10359251; doi:10.1038/s41467-023-40122-2)
Supplement: Supplementary file 1 — Supplementary Information [file 41467_2023_40122_MOESM1_ESM.pdf]

Supplementary Information

Polarization Conversion in Bottom-up Grown Quasi-1D Fibrous Red Phosphorus Flakes

Zhaojian Sun[1,2], Wujia Chen[1,2], Bowen Zhang[1], Lei Gao[1], Kezheng Tao[1], Qiang Li[1], Jia-Lin Sun[3], and Qingfeng Yan[1,*]

[1]Engineering Research Center of Advanced Rare Earth Materials, Ministry of Education, Department of Chemistry, Tsinghua University, Beijing 100084, P. R. China.

[2]These authors contributed equally: Zhaojian Sun, Wujia Chen.

[3]Department of Physics, Tsinghua University, Beijing 100084, P. R. China.

*Corresponding author: yanqf@mail.tsinghua.edu.cn.

Supplementary Results

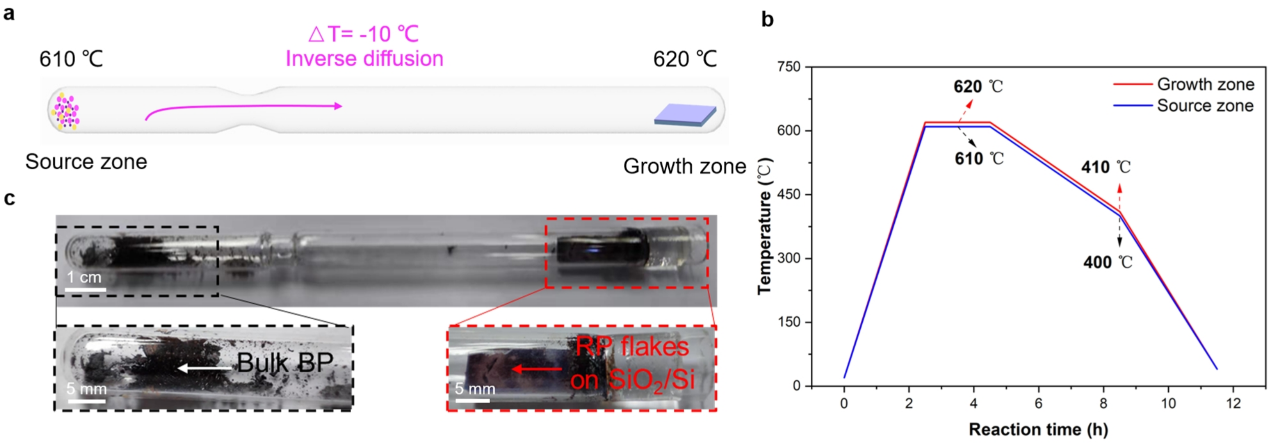

Supplementary Fig. 1 a Schematic illustration of CVT growth of fibrous RP flakes on the substrate under inverse temperature gradient of $\Delta T = -10$ ℃. **b** Corresponding CVT reaction temperature programs. **c** Photos of resulted products after CVT reaction. Bulk BP crystals appear in the source zone while fibrous RP flakes grow on the SiO_2/Si substrate in the growth zone.

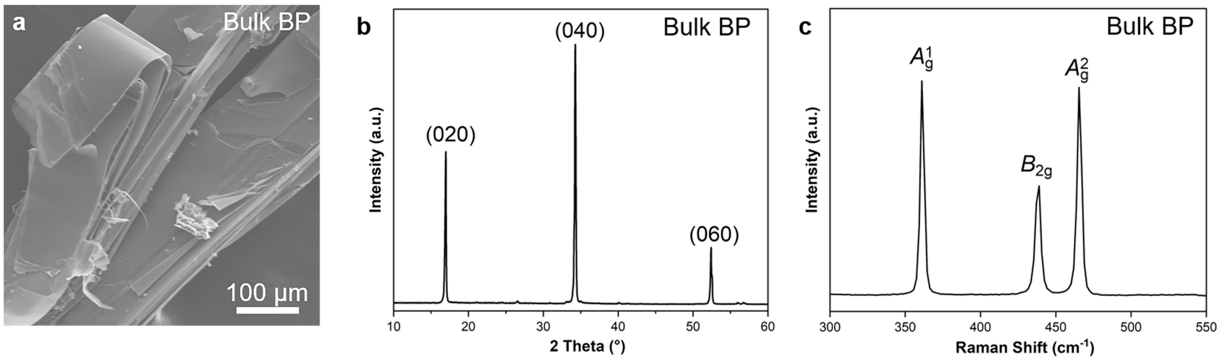

Supplementary Fig. 2 **a** Typical SEM image, **b** XRD pattern, and **c** Raman spectrum of bulk BP crystals. The unit of intensity is arbitrary units (a.u.).

SEM image in Supplementary Fig. 2a shows clear layered structure characteristic for BP bulk crystals. XRD pattern in Supplementary Fig. 2b exhibits typical BP diffraction peaks from the (020), (040), and (060) planes located at 17.0º, 34.2º and 52.1º, respectively. Supplementary Fig. 2c demonstrates BP Raman peaks located at 361.1, 438.5 and 465.4 cm^{-1}, assigned to A_g^1, B_{2g}, and A_g^2 phonon modes. The above characterizations confirm the formation of bulk BP single crystals in the source zone shown in Supplementary Fig. 1c.

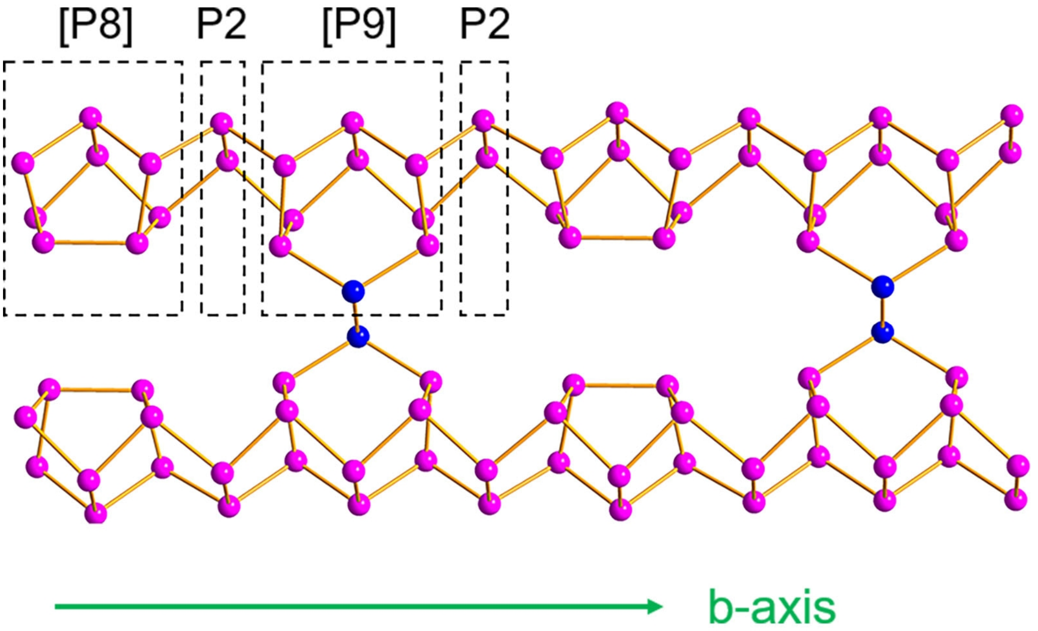

Supplementary Fig. 3 Crystal structure of fibrous RP, showing the building units of [P8]P2[P9]P2[.

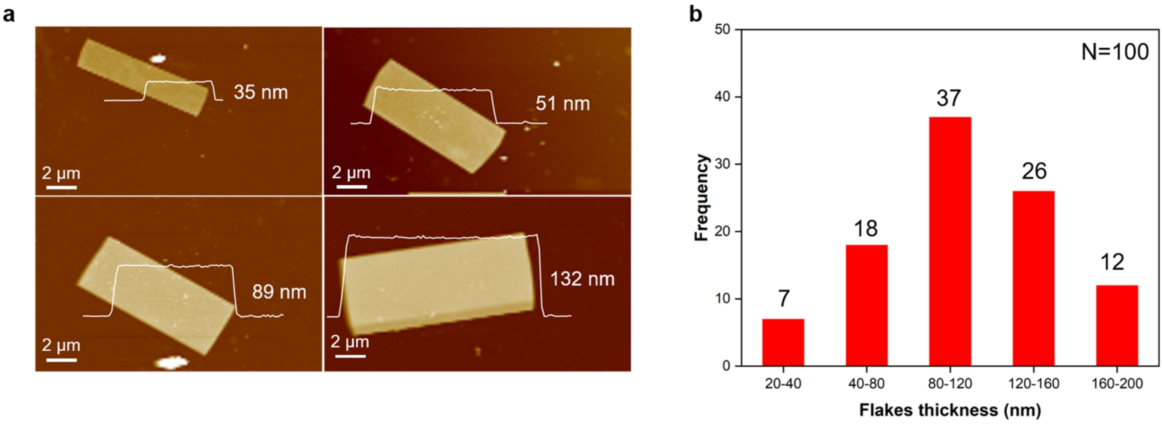

Supplementary Fig. 4 **a** More AFM images of fibrous RP flake with different thicknesses grown on the SiO_2/Si substrate. **b** The thickness distribution of fibrous RP flakes. The number of randomly selected flakes is 100.

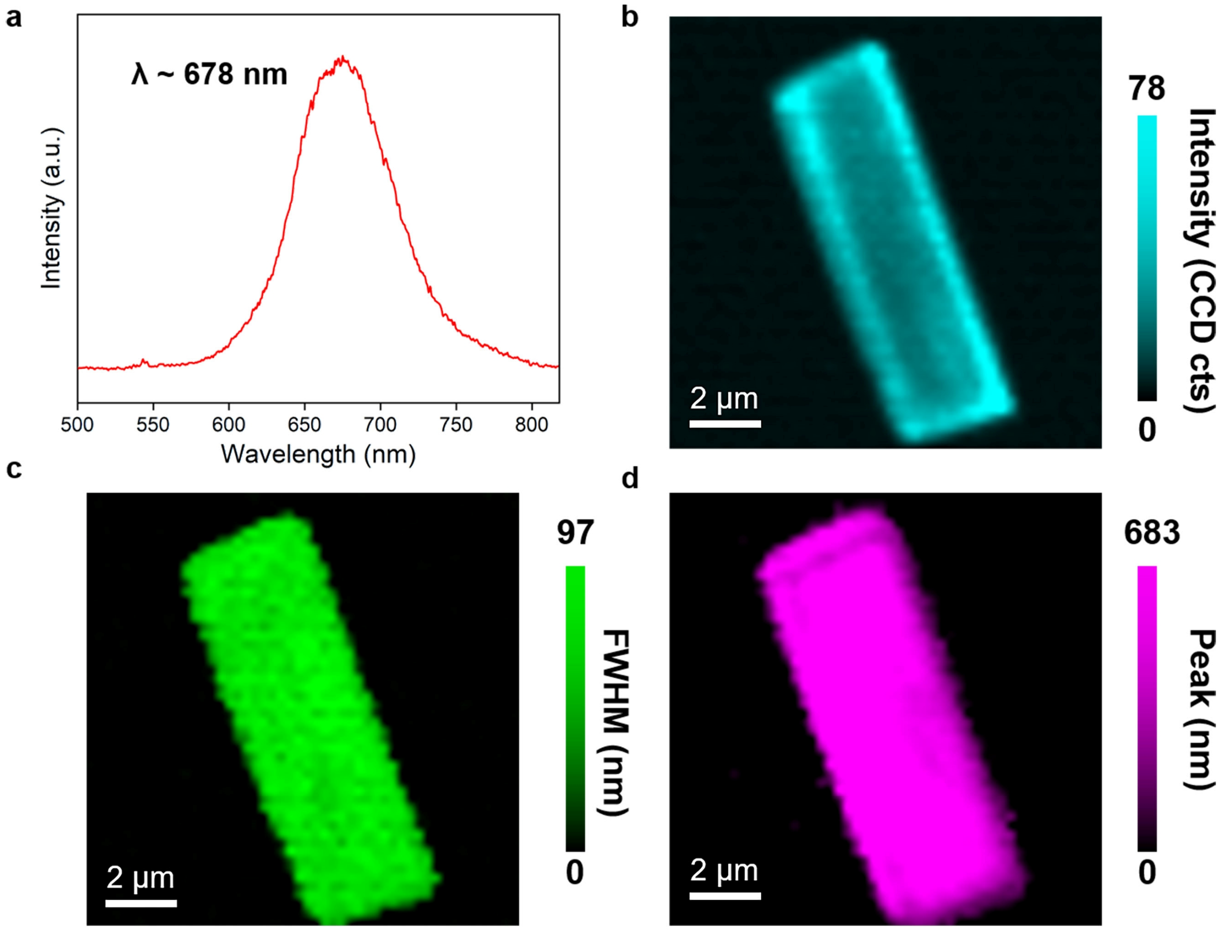

Supplementary Fig. 5 **a** PL spectrum of the fibrous RP flake. **b-d** The mapping of photoluminescence intensity, FWHM and peak, respectively**.**

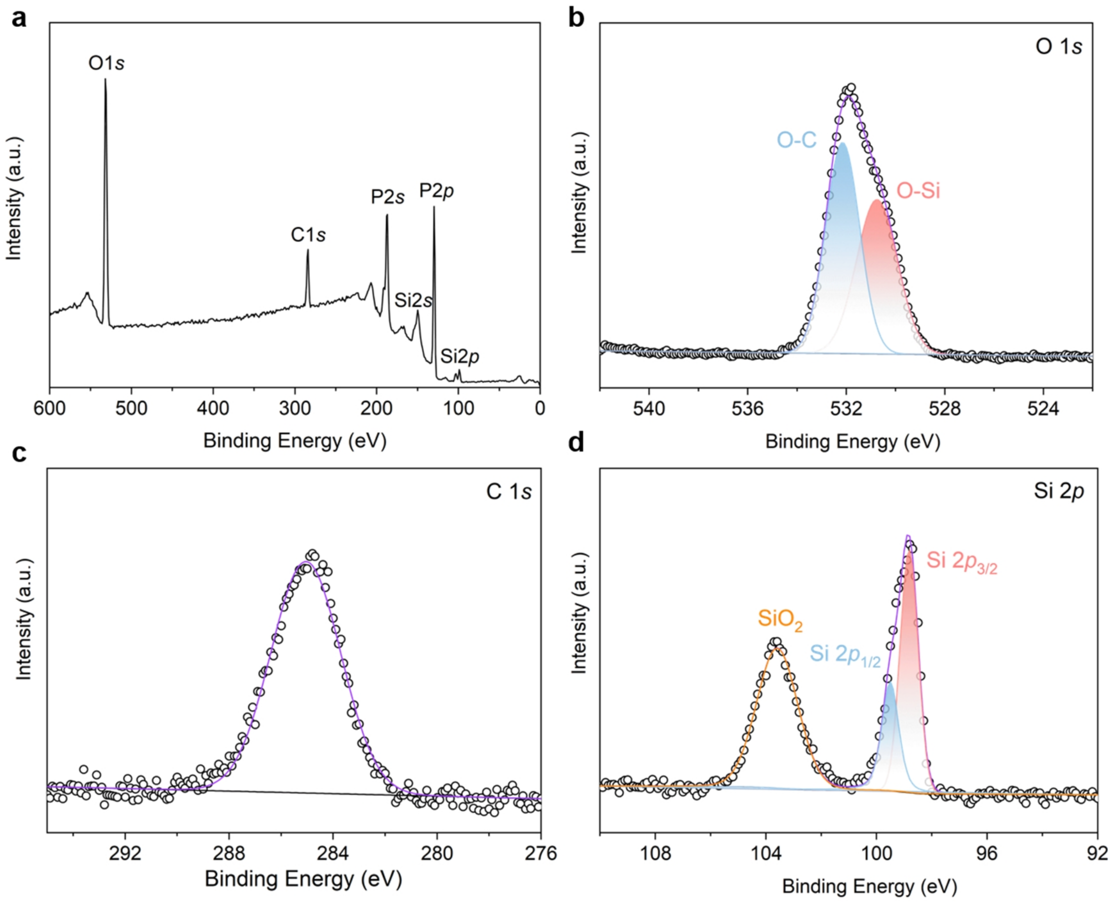

Supplementary Fig. 6 a Survey XPS spectrum. High-resolution XPS spectra of **b** O 1*s*, **c** C 1*s*, **d** Si 2*p* for fibrous RP flakes directly grown on the SiO_2/Si substrate.

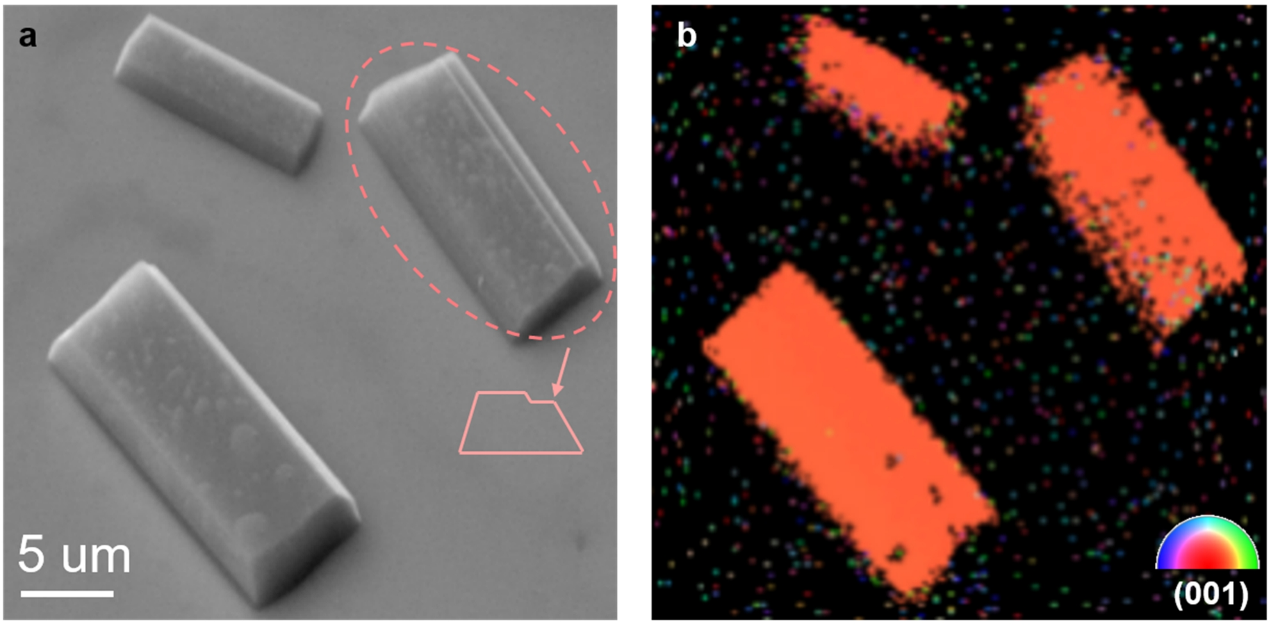

Supplementary Fig. 7 **a** SEM image of the fibrous RP flakes grown on the SiO_2/Si substrate. **b** The corresponding inverse pole figure (IPF) along the z-axis, demonstrating the distinct (001) crystal orientation.

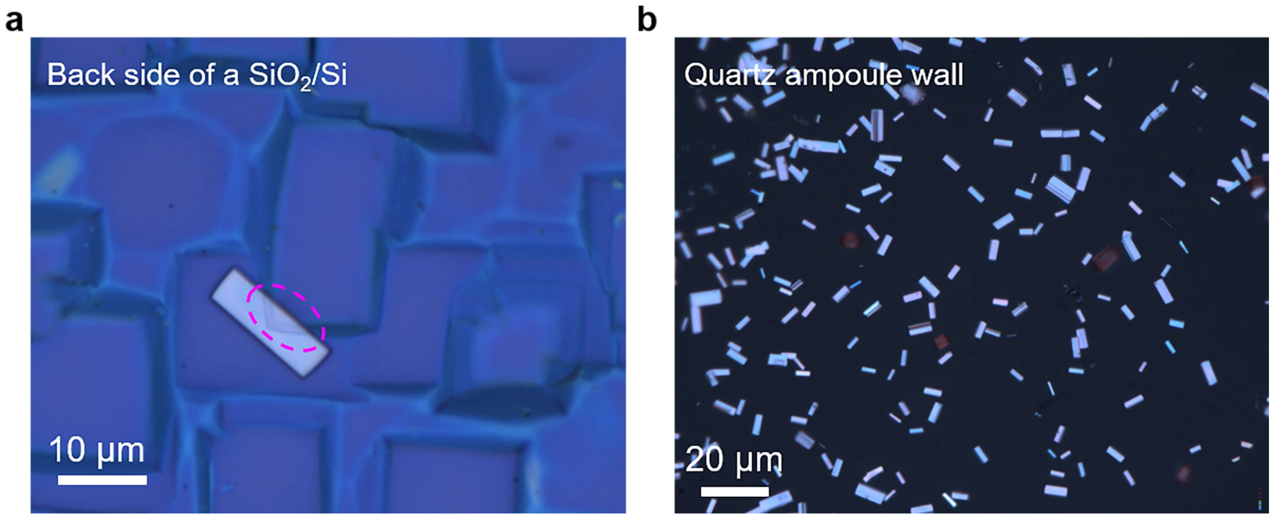

Supplementary Fig. 8 The representative optical image of a fibrous RP flake directly grown on **a** the back side of a SiO_2/Si substrate and **b** the inner wall of the quartz ampoule. The red dashed ellipse highlights that the fibrous RP flake could crystallize and grow across a step edge.

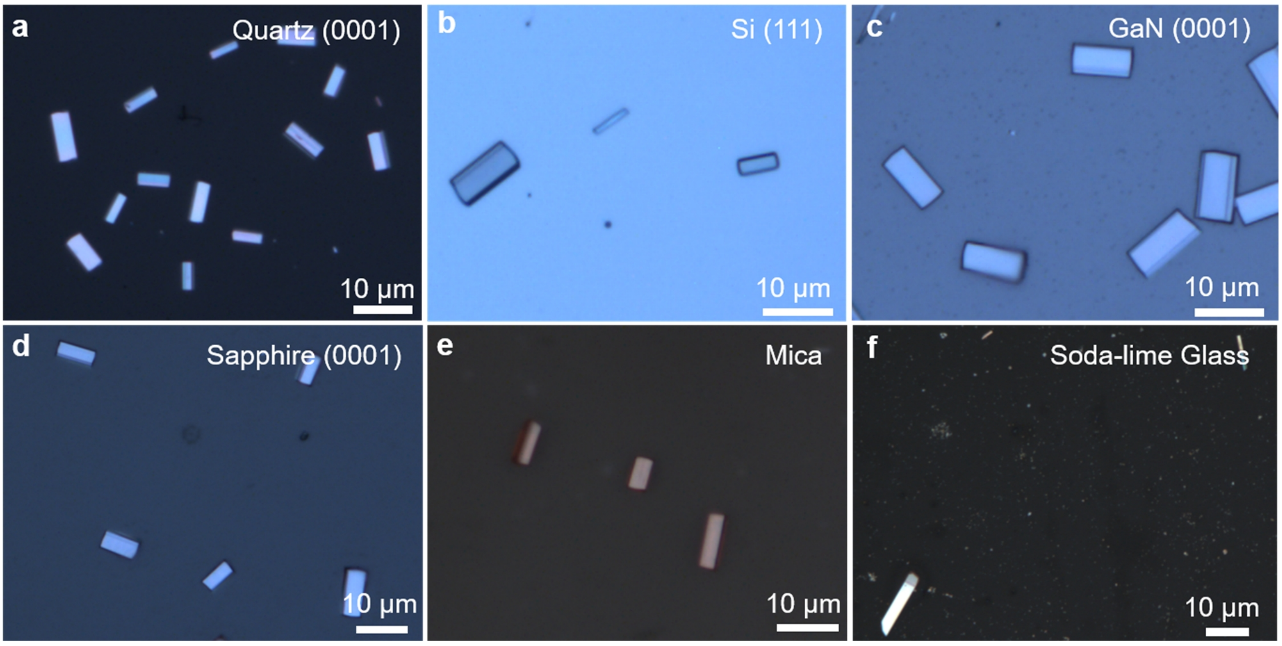

Supplementary Fig. 9 Optical images of fibrous RP flakes directly grown on diverse substrates of **a** quartz (0001), **b** Si (111), **c** GaN (0001), **d** sapphire (0001), **e** mica, and **f** soda-lime glass.

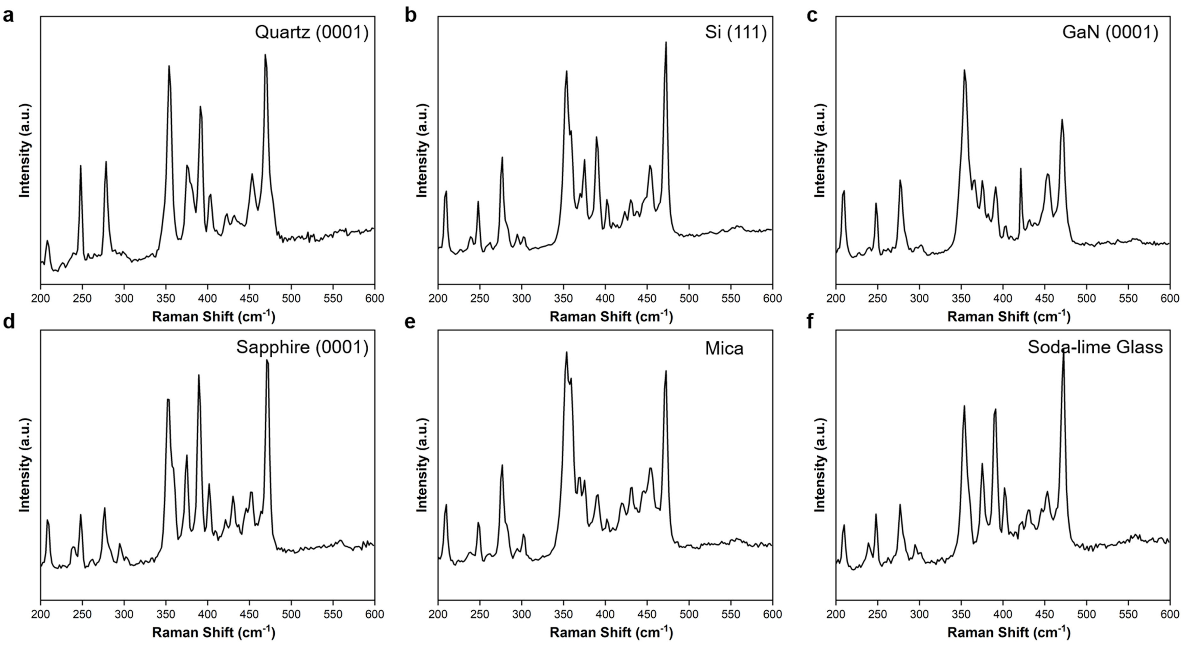

Supplementary Fig. 10 Raman spectra of the fibrous RP flake directly grown on the **a** quartz (0001), **b** Si (111), **c** GaN (0001), **d** sapphire (0001), **e** mica, and **f** soda-lime glass.

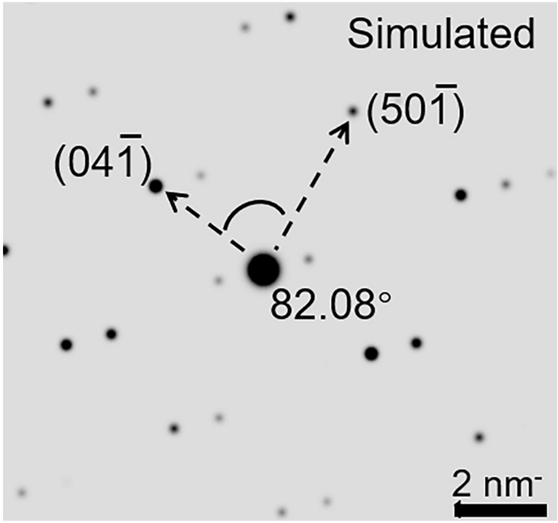

Supplementary Fig. 11 The simulated electron diffraction pattern viewed along the zone axis $[\bar{4}\ \bar{5}\ \overline{20}]$ for fibrous RP by using Crystal Maker software.

Supplementary Table 1 Surface energy of (100), (010), and (001) planes for fibrous RP.

Crystallographic plane	(100)	(010)	(001)
Surface energy [meV·Å^{-2}]	10.10	33.60	8.27

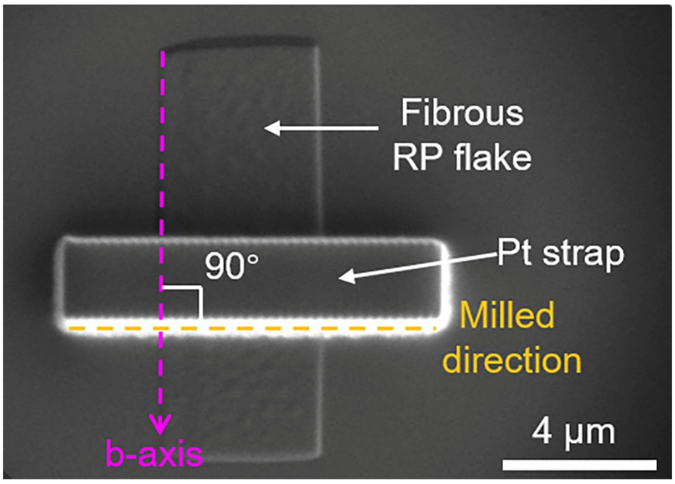

Supplementary Fig. 12 The preparation of cross-sectional STEM sample by FIB. The milled direction is perpendicular to the long parallel sides (b-axis) of the fibrous RP flake.

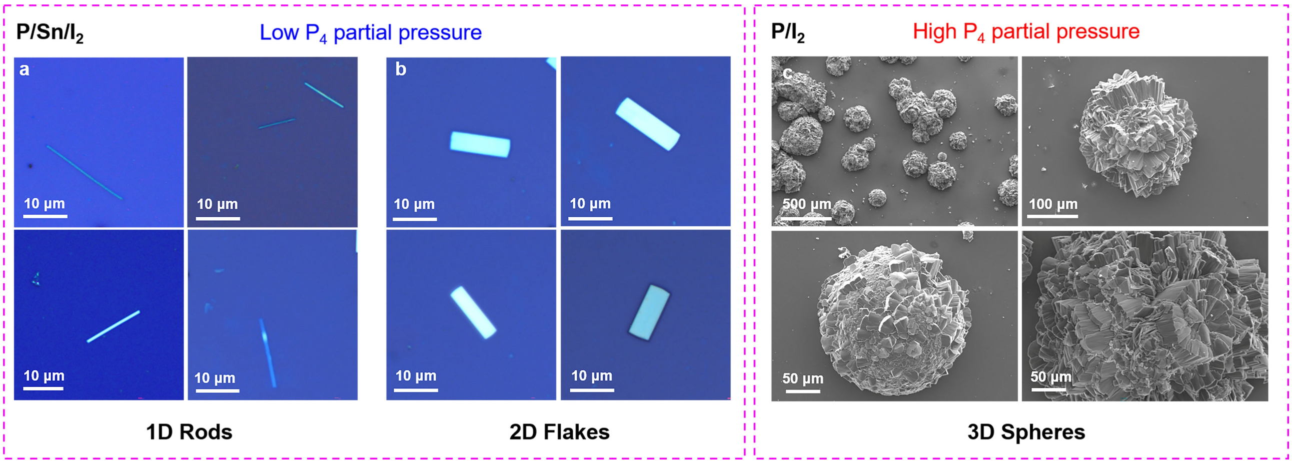

Supplementary Fig. 13 Morphology evolution of fibrous RP under low/high P_4 partial pressure in the $P/Sn/I_2$ and P/I_2 system, respectively.

Supplementary Table 2. Energy values in Hartree derived from DFT calculations for all structures at different temperatures.

**local minimum structures**	E_{tot}
P_4	-1364.648935
SnI_2	-26.3428969
I_2	-22.8481693
**i**	-6246.542428
**ii**	-14540.22618
**iii**	-14329.30335

Supplementary Table 3. Differential total energies ΔE_{tot} and DG for local minimum structures **i** to **iii**, referenced to the starting materials. (Unit: kJ/mol)

**local minimum structures**	ΔE_{tot}
**i**	-658.0
**ii**	-1757.0
**iii**	-1285.3

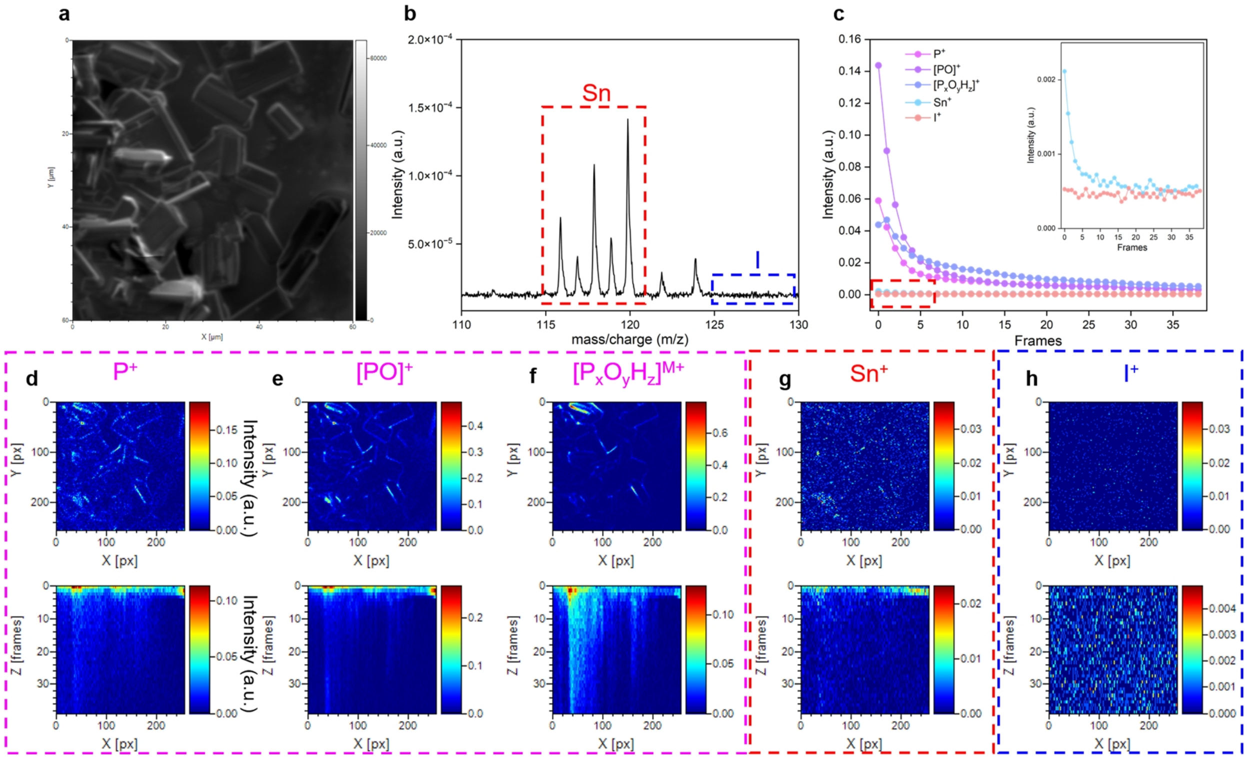

Supplementary Fig. 14 a SEM images of the selected area. **b** Mass spectra of tin and iodine. **c** Depth profile of the P^+, $[PO]^+$, $[P_xO_yH_z]^{M+}$, Sn^+ and I^+ within the selected area. Inset: an enlargement of the red dotted rectangle. The surface and cross-sectional ion analysis mapping of **d** P^+, **e** $[PO]^+$, **f** $[P_xO_yH_z]^{M+}$, **g** Sn^+ and **h** I^+ measured by ToF-SIMS. Top: surface ion analysis mapping. Down: cross-sectional ion analysis mapping.

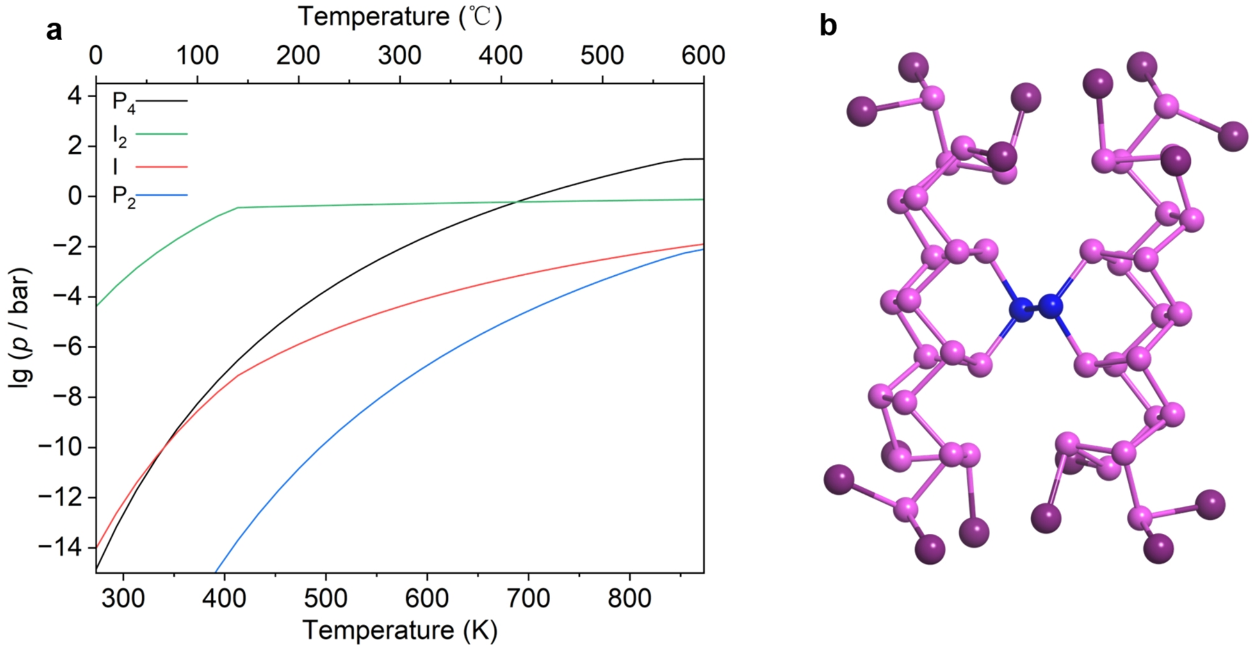

Supplementary Fig. 15 a Partial pressure diagram of the corresponding equilibrium gas pressure of P/I_2 system in the temperature ranges from 273 K t o873 K calculated by CalPhaD **b** Schematic diagram of the geometry-optimized structures of fibrous RP capped with single iodine atom.

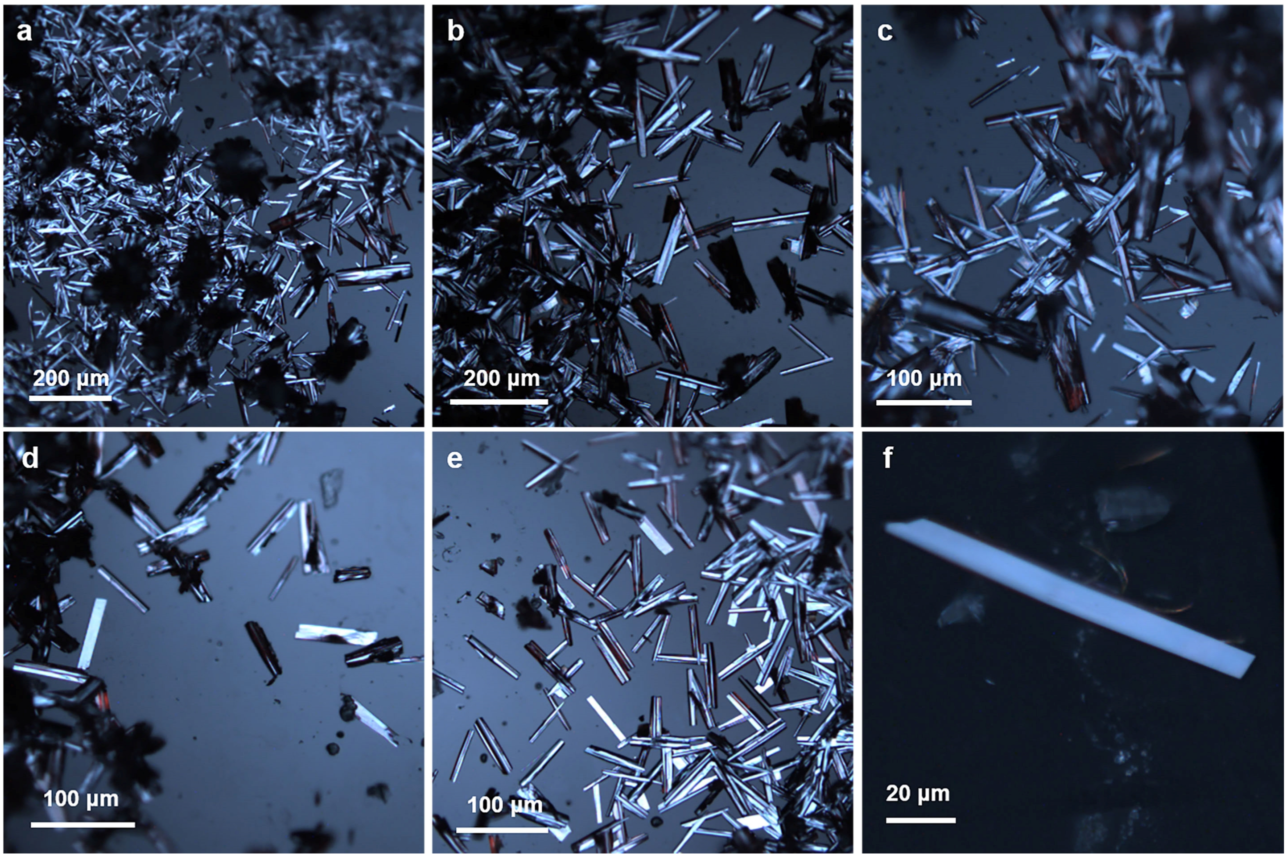

Supplementary Fig. 16 Optical images of the fibrous RP flakes with high aspect ratio obtained in the improved P/I_2 system.

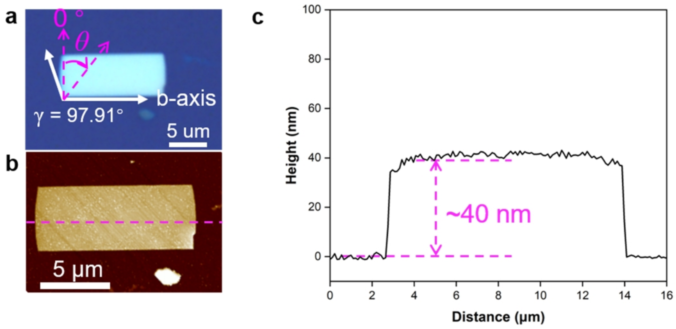

Supplementary Fig. 17 **a** The optical image of fibrous RP flake on the SiO_2/Si. 0° is selected as the initial direction of incident laser, which is perpendicular to the b-axis of fibrous RP. We defined the angle between the incident light polarization direction and 0° as θ. **b** AFM image, and **c** corresponding line profiles of a fibrous RP flake with a thickness of 40 nm.

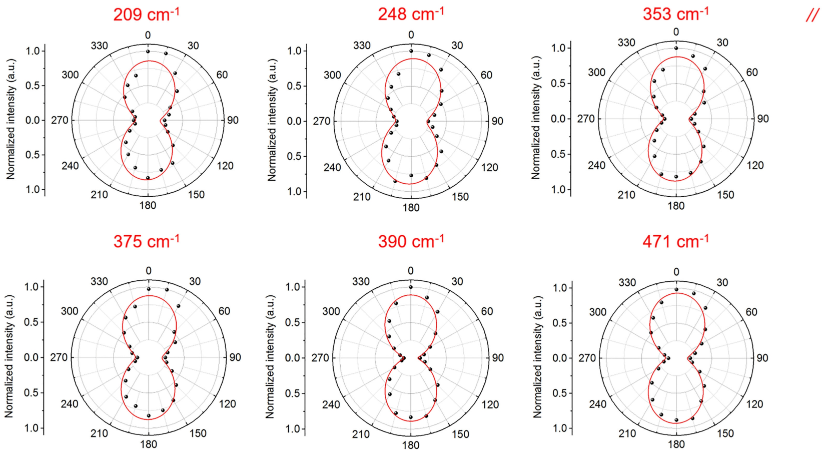

Supplementary Fig. 18 Polar plots for the different characteristic Raman peaks of the fibrous RP flake with a thickness of 40 nm under 532 nm excitation and parallel-polarization configuration. The fitted red curves match well with the experimental data (black dots).

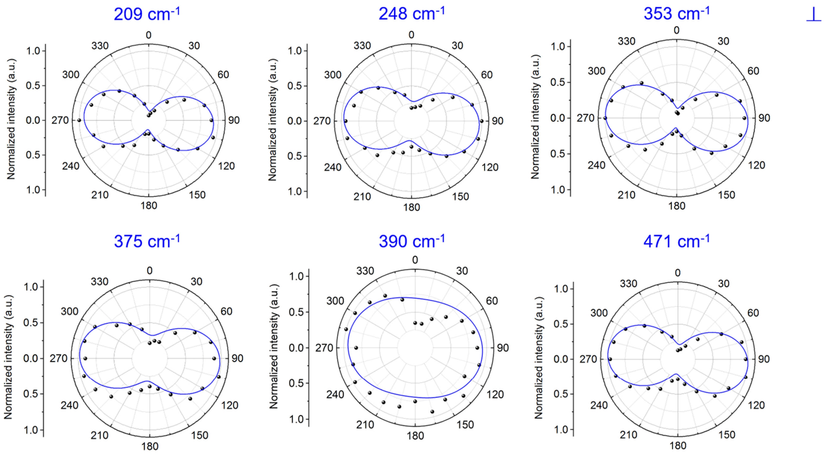

Supplementary Fig. 19 Polar plots for the different characteristic Raman peaks of the fibrous RP flake with a thickness of 40 nm under 532 nm excitation and cross-polarization configuration. The fitted blue curves match well with the experimental data (black dots).

To quantitatively analyze the polarization-dependent Raman spectra, Raman scattering analysis is employed. The intensity of Raman scattering can be described by the following equation[1,2]:

$$\propto \sum_j \left|\mathbf{e_s} \cdot \mathbf{R_j} \cdot \mathbf{e_i}\right|^2, \tag{1}$$

where $\mathbf{R_j}$ is a 3×3 Raman tensor, $\mathbf{e_i}$ and $\mathbf{e_s}$ represent the unit polarization vectors of the incident laser and scattered Raman signal, respectively. The polarization vector for incident light is $\mathbf{e_i}^{\mathrm{T}}$= (sinθ cosθ 0), while $\mathbf{e_s}$ = (0 1 0) under the parallel-configuration and $\mathbf{e_s}$ = (1 0 0) under the cross-configuration. Fibrous RP belongs to C_i point group, and its Raman tensor can be expressed as[3]:

$$\mathrm{R}(A_g)=\begin{pmatrix} \boldsymbol{a} & \boldsymbol{d} & \boldsymbol{e} \\ \boldsymbol{d} & \boldsymbol{b} & \boldsymbol{f} \\ \boldsymbol{e} & \boldsymbol{f} & \boldsymbol{c} \end{pmatrix} . \tag{2}$$

Hence, the observed anisotropic Raman scattering intensity for A_g mode can be expressed by the following equations[1]:

$$I\,(A_{g,} /\!/) = (\boldsymbol{b}\cos\theta + \boldsymbol{d}\sin\theta)^2, \tag{3}$$

$$I\,(A_{g,} \perp) = (\boldsymbol{d}\cos\theta + \boldsymbol{a}\sin\theta)^2. \tag{4}$$

As observed in Supplementary Figs. 18, 19, the fitted red and blue curves match well with the experimental data (black dots). Due to $|\boldsymbol{b}/\boldsymbol{d}| > 1$ and $|\boldsymbol{a}/\boldsymbol{d}| > 1$, the direction along the maximum Raman intensity in polar plots is perpendicular to the b-axis for parallel-configuration, while parallel to the b-axis for cross-configuration.

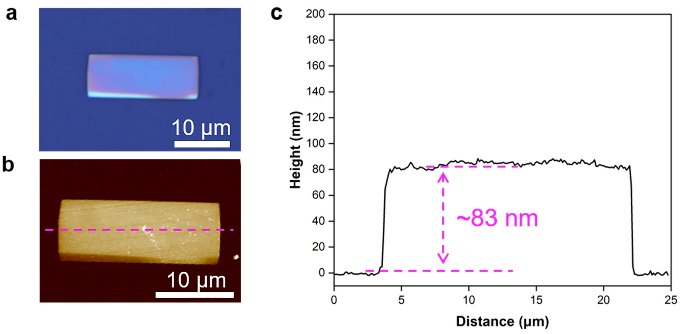

Supplementary Fig. 20 a The optical image, **b** AFM image, and **c** corresponding line profiles of a fibrous RP flake with a thickness of 83 nm.

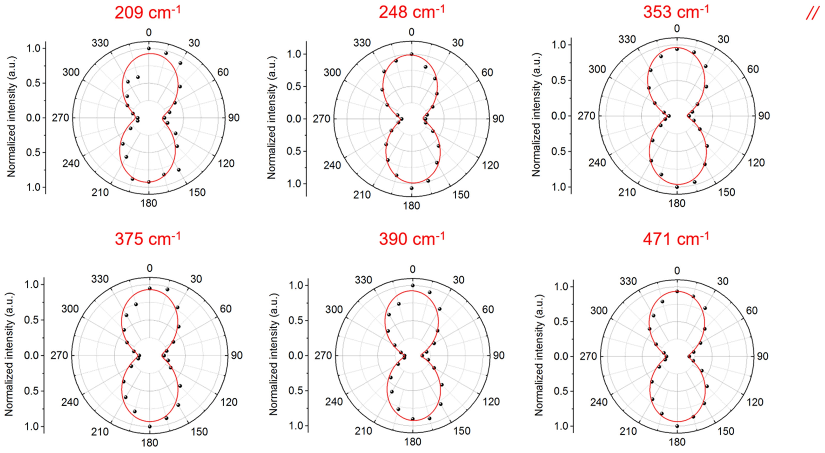

Supplementary Fig. 21 Polar plots for the different characteristic Raman peaks of the fibrous RP flake with a thickness of 83 nm under 532 nm excitation and parallel-polarization configuration. The fitted red curves match well with the experimental data (black dots).

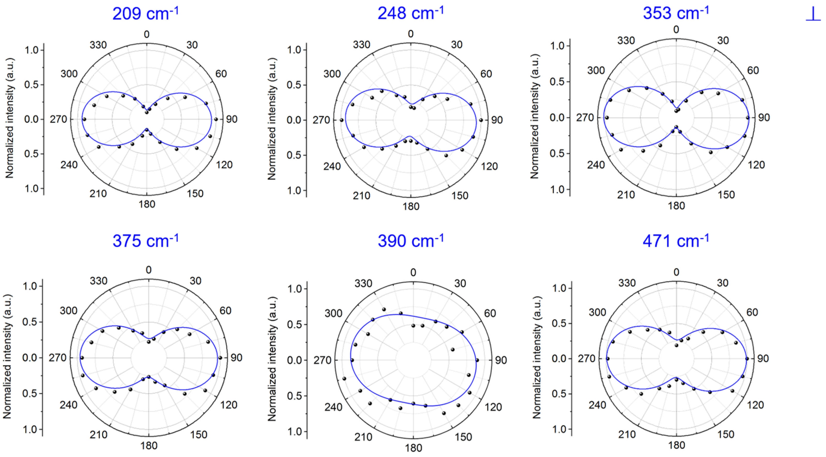

Supplementary Fig. 22 Polar plots for the different characteristic Raman peaks of the fibrous RP flake with a thickness of 83 nm under 532 nm excitation and cross-polarization configuration. The fitted blue curves match well with the experimental data (black dots).

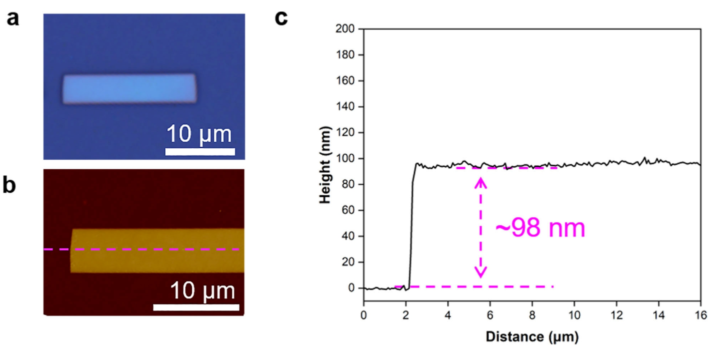

Supplementary Fig. 23 a The optical image, **b** AFM image, and **c** corresponding line profiles of a fibrous RP flake with a thickness of 98 nm.

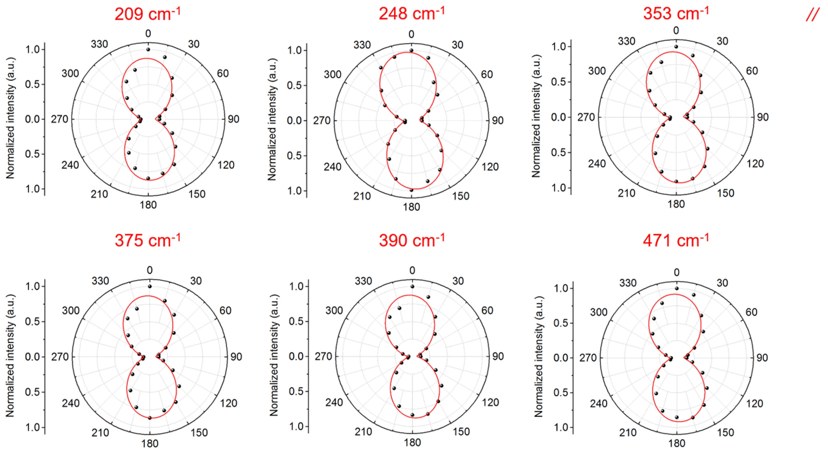

Supplementary Fig. 24 Polar plots for the different characteristic Raman peaks of the fibrous RP flake with a thickness of 98 nm under 532 nm excitation and parallel-polarization configuration. The fitted red curves match well with the experimental data (black dots).

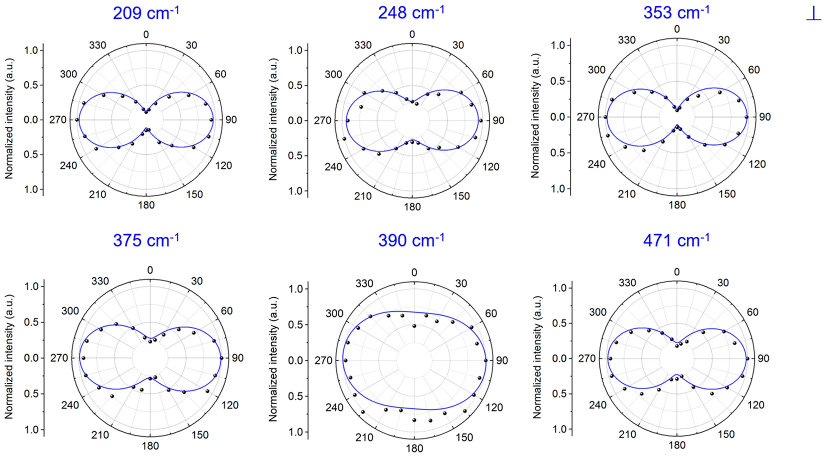

Supplementary Fig. 25 Polar plots for the different characteristic Raman peaks of the fibrous RP flake with a thickness of 98 nm under 532 nm excitation and cross-polarization configuration. The fitted blue curves match well with the experimental data (black dots).

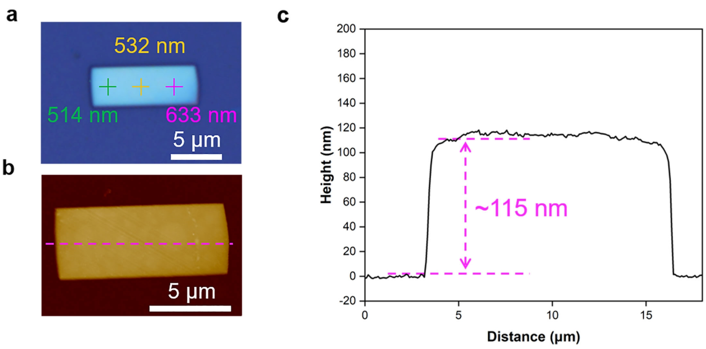

Supplementary Fig. 26 **a** The optical image, **b** AFM image, and **c** corresponding line profiles of a fibrous RP flake with a thickness of 115 nm. Three locations marked by crosses in Supplementary Fig. 26a were selected to measure angle-resolved polarized Raman signals under various excitation lasers of 514 nm, 532 nm and 633 nm.

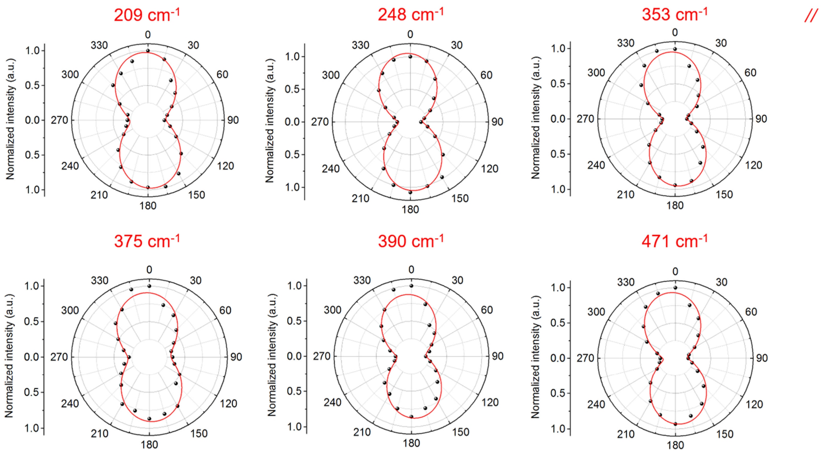

Supplementary Fig. 27 Polar plots for the different characteristic Raman peaks of the fibrous RP flake with a thickness of 115 nm under 514 nm excitation and parallel-polarization configuration. The fitted red curves match well with the experimental data (black dots).

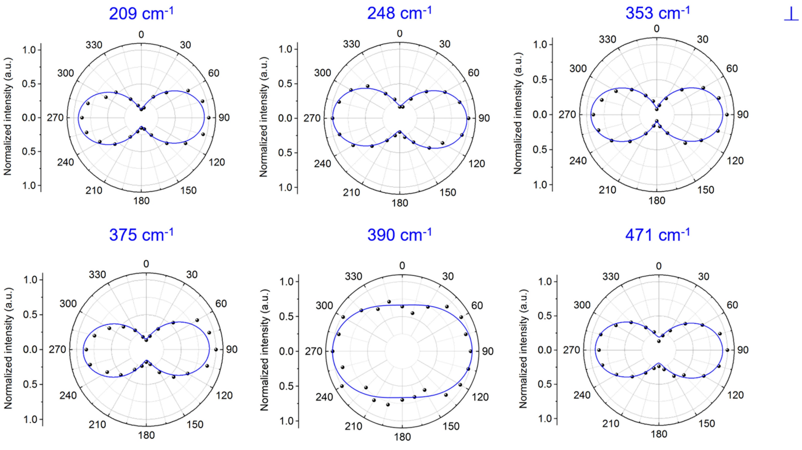

Supplementary Fig. 28 Polar plots for the different characteristic Raman peaks of the fibrous RP flake with a thickness of 115 nm under 514 nm excitation and cross-polarization configuration. The fitted blue curves match well with the experimental data (black dots).

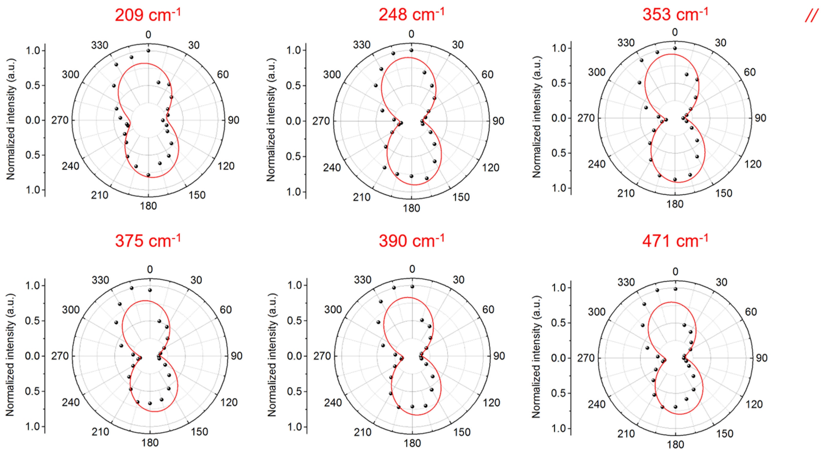

Supplementary Fig. 29 Polar plots for the different characteristic Raman peaks of the fibrous RP flake with a thickness of 115 nm under 532 nm excitation and parallel-polarization configuration. The fitted red curves match well with the experimental data (black dots).

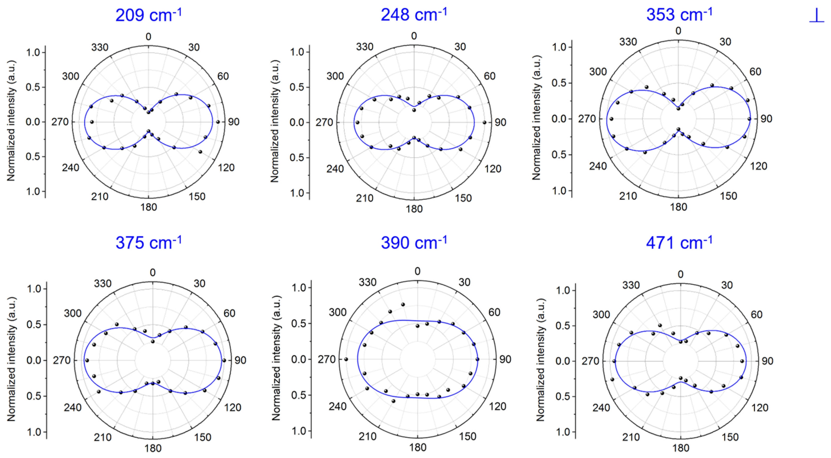

Supplementary Fig. 30 Polar plots for the different characteristic Raman peaks of the fibrous RP flake with a thickness of 115 nm under 532 nm excitation and cross-polarization configuration. The fitted blue curves match well with the experimental data (black dots).

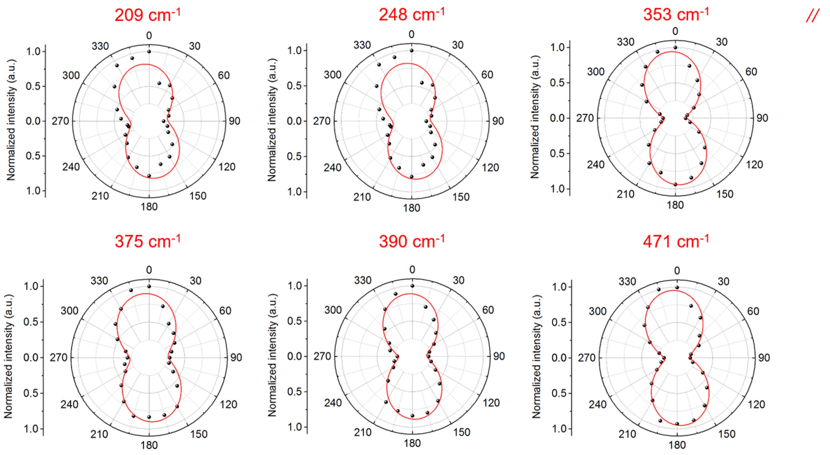

Supplementary Fig. 31 Polar plots for the different characteristic Raman peaks of the fibrous RP flake with a thickness of 115 nm under 633 nm excitation and parallel-polarization configuration. The fitted red curves match well with the experimental data (black dots).

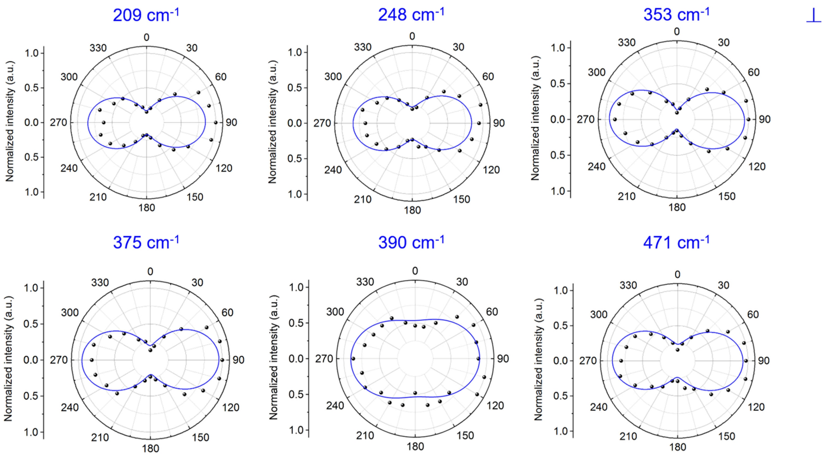

Supplementary Fig. 32 Polar plots for the different characteristic Raman peaks of the fibrous RP flake with a thickness of 115 nm under 633 nm excitation and cross-polarization configuration. The fitted blue curves match well with the experimental data (black dots).

Supplementary Table 4 Polar plots for the typical Raman mode (471 cm^{-1}) of fibrous RP flakes under two polarization configurations **a** with different flake thicknesses under 532 nm excitation, **b** under various excitation laser wavelengths with a thickness of 115 nm.

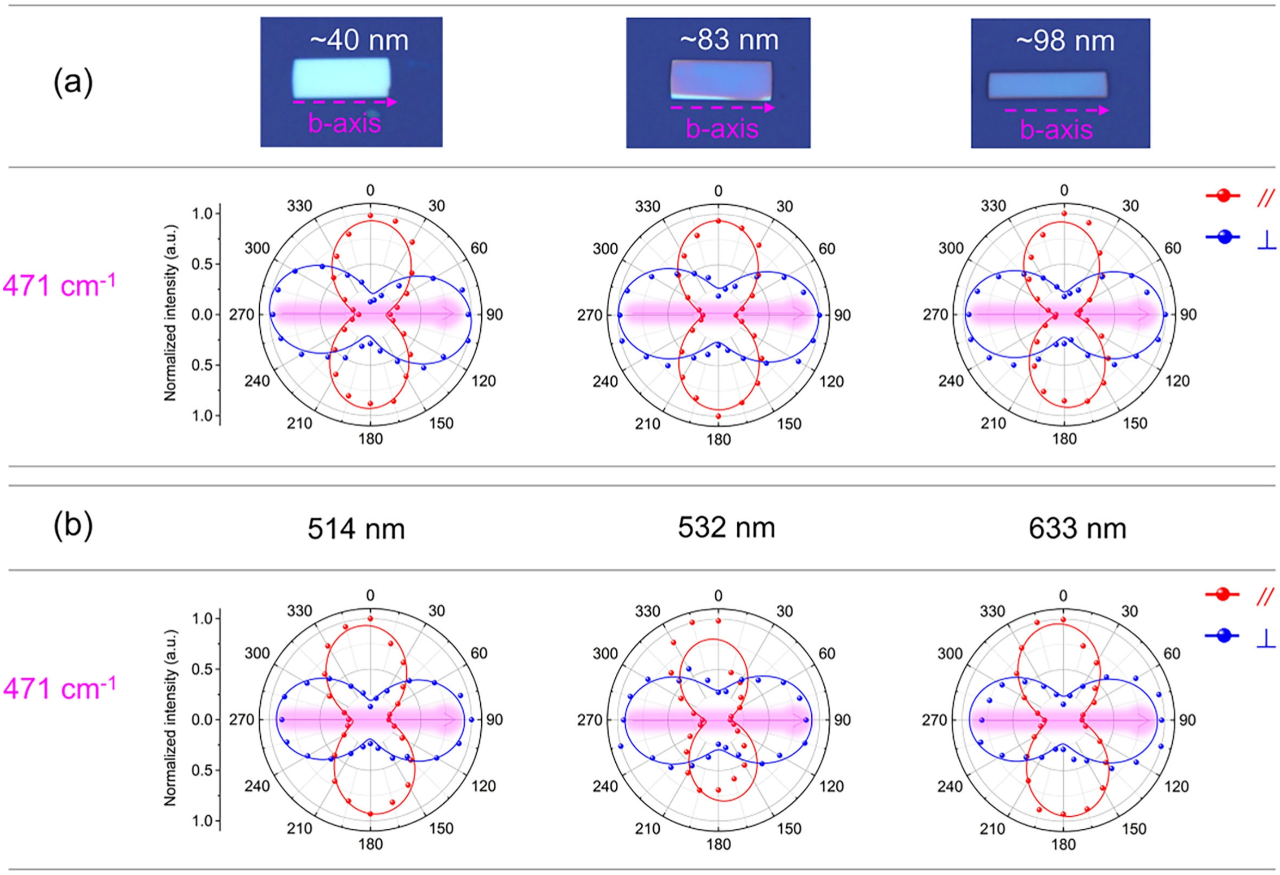

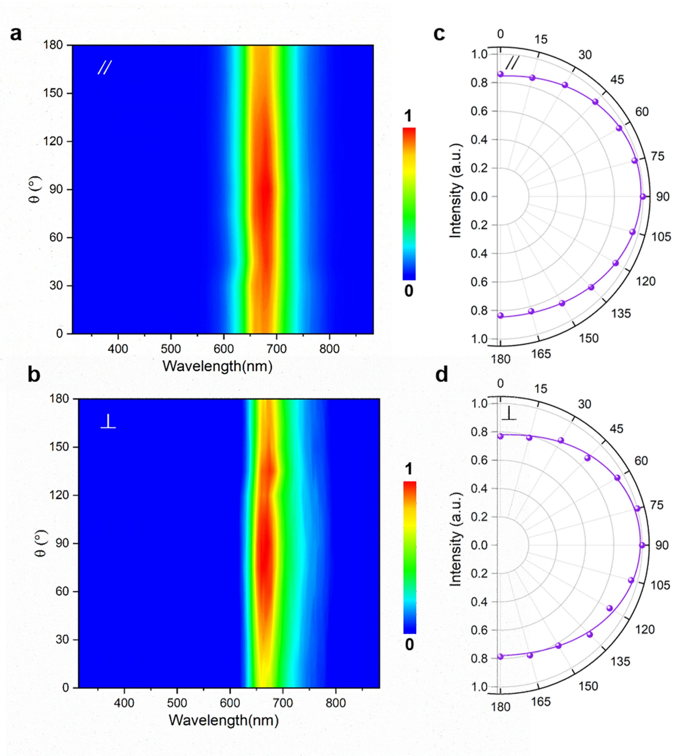

Supplementary Fig. 33 Contour plot of polarized PL intensity under **a** parallel (//) and **b** cross (⊥) polarization configuration. Polar plots of the polarized PL intensity under **c** parallel (//) and **d** cross (⊥) polarization configuration.

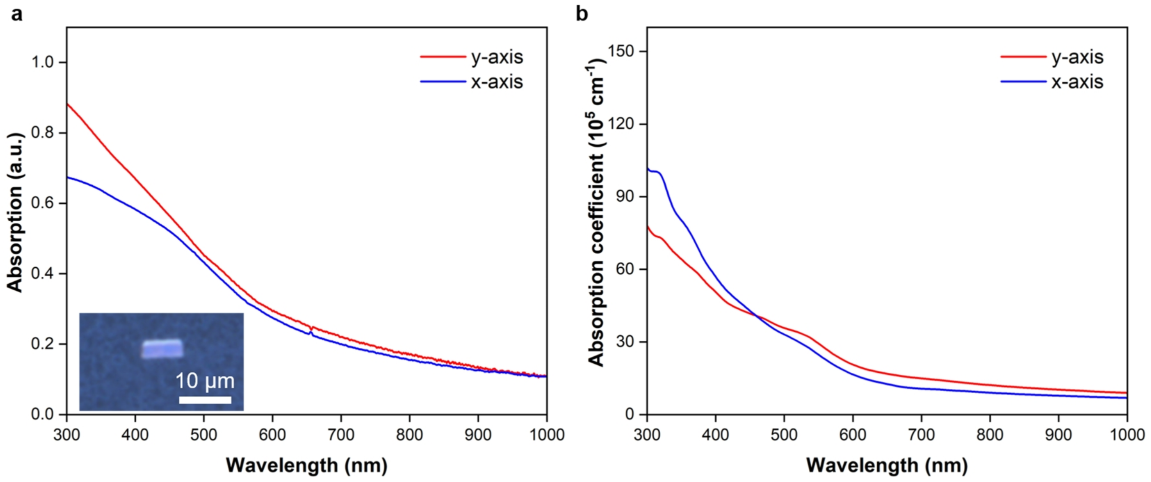

Supplementary Fig. 34 **a** Polarized micro-area UV-vis-NIR optical absorption spectra along x-axis and y-axis. Inset: The optical image of fibrous RP flake on the transparent mica substrate for measurement. **b** Absorption coefficient of bulk fibrous RP along x-axis and y-axis calculated by density functional theory.

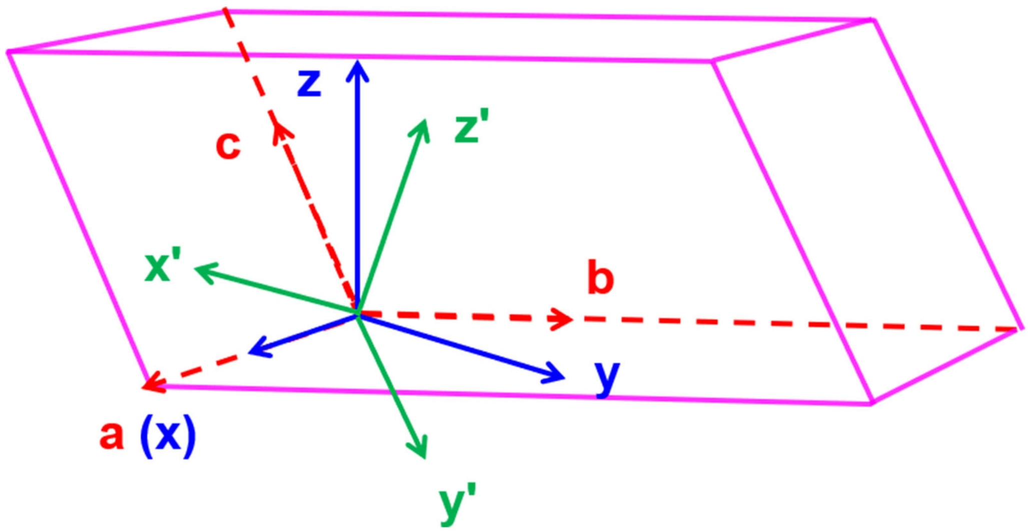

Supplementary Fig. 35 Schematic illustration of crystallographic coordinate system, rectangular coordinate system and optical principal axis coordinate system. Crystallographic coordinate system (red): a-axis, b-axis and c-axis. Rectangular coordinate system (blue): x-axis, y-axis, z-axis. Optical principal axis coordinate system (green): x'-axis, y'-axis, z'-axis.

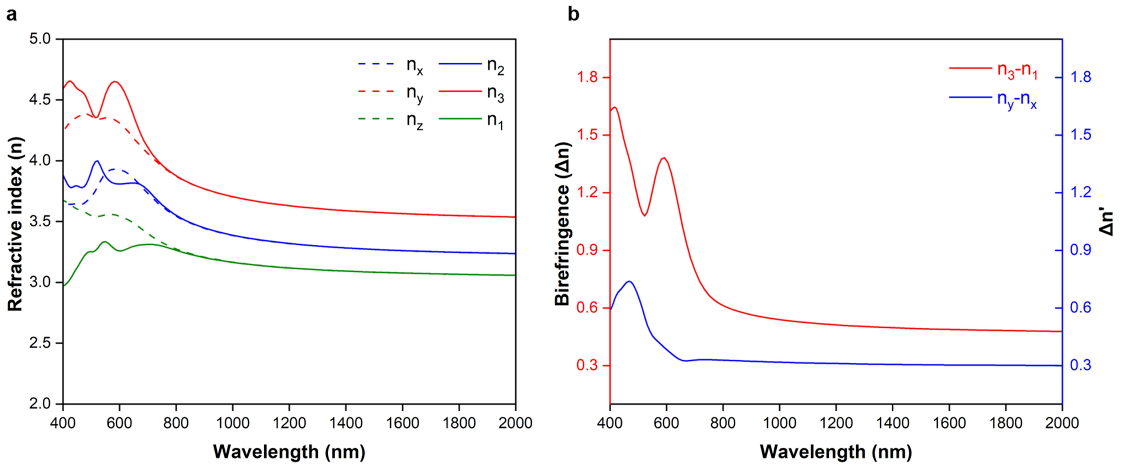

Supplementary Fig. 36 **a** Calculated dispersion relation of refractive index n along different axes for fibrous RP. **b** Calculated birefringence (Δn) and in-plane refractive index difference ($\Delta n'$) for wavelengths from 400 to 2000 nm.

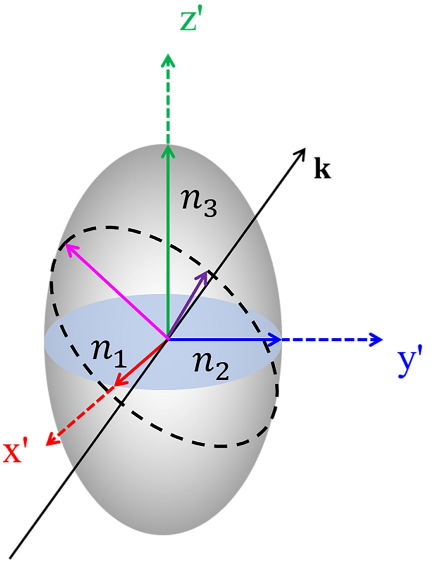

Supplementary Fig. 37 Schematic illustration of refractive ellipsoid for triclinic crystal.

The x', y', z' and n_1, n_2, n_3 represent the optical principal axes and principal refractive index, respectively. When the light vector is incident along the wave normal **k**, the cross section obtained by intersecting the ellipsoid and the vertical plane of the wave normal **k** passing through the origin is an ellipse. The semi-major (pink) and semi-minor axes (purple) of the ellipse are the slow and fast axes, respectively.

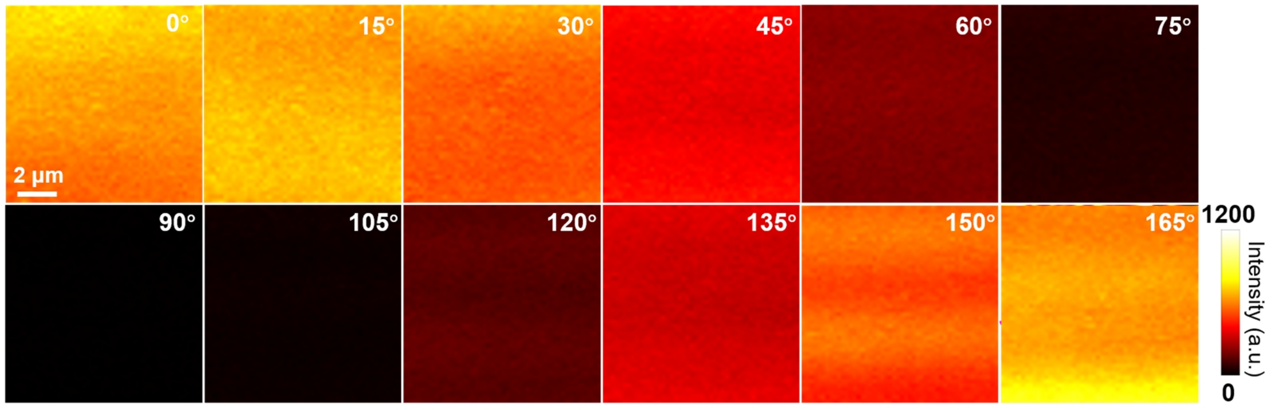

Supplementary Fig. 38 Polarization-resloved monochromatic transmitted light intensity mappings of the transparent quartz substrate at different analyzer angles (Analyzer angle from 0° to 165°, with a step size of 15°).

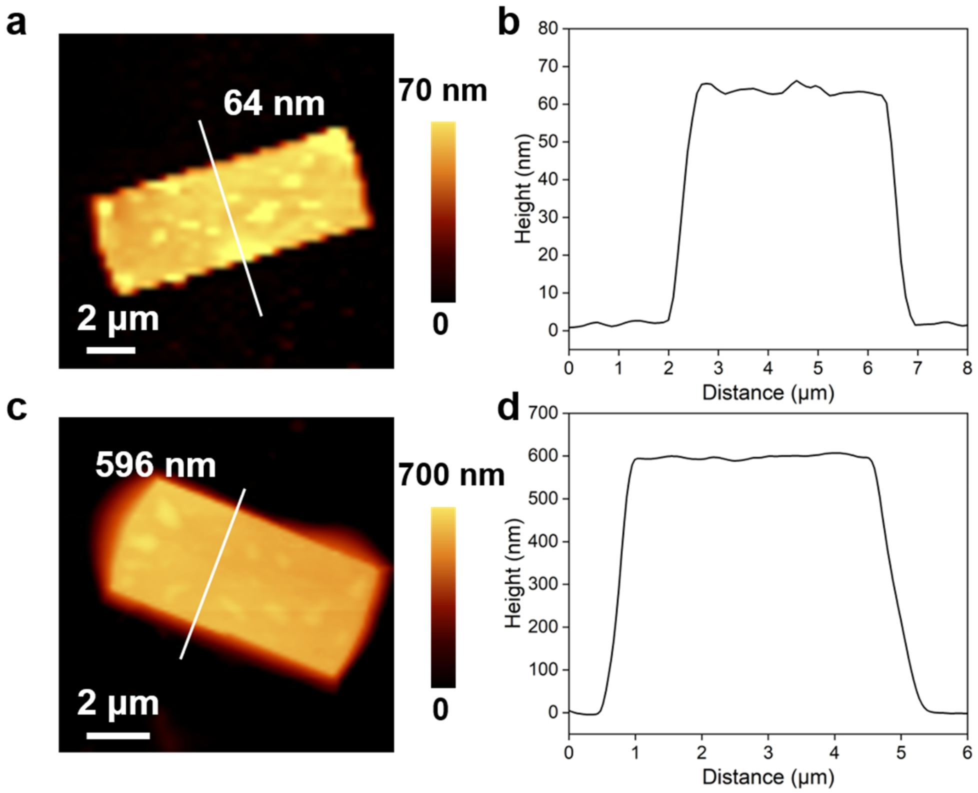

Supplementary Fig. 39 a, c AFM images, and **b,d** the corresponding line profiles of the fibrous RP flakes with a thickness of 64 nm and 596 nm, respectively.

To further quantitatively explain the influencing factors of DOP value, the following derivation is employed.

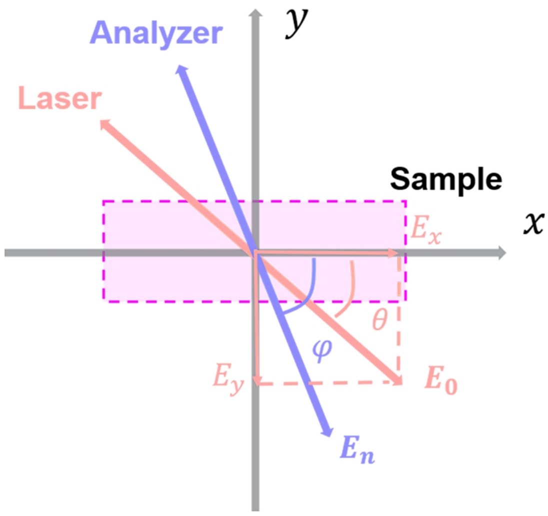

Supplementary Fig. 40 Schematic diagram of the light vector decomposition.

As depicted in Supplementary Fig. 40, the incident light vector can be decomposed into components along the fast and slow axes of the fibrous RP. In order to streamline the model, we adopt the approximation that the b-axis of the fibrous RP represents the fast-axis. Consequently, upon traversing the fibrous RP flakes, the transmitted light vector can be expressed as follows:

$$\mathbf{E} = E_0 \cdot cos\,\theta \cdot t_x \cdot cos(\omega t) \cdot \mathbf{x} + E_0 \cdot sin\,\theta \cdot t_y \cdot cos(\omega t - \delta) \cdot \mathbf{y} \tag{5}$$

The transmitted light vector along the fast and slow axes can be further decomposed along the direction of analyzer. The transmitter light vector after passing the analyzer can be expressed as follow:

$$\mathbf{E_n} = [E_0 \cdot cos\,\theta \cdot t_x \cdot cos\,\varphi \cdot \cos(\omega t) + E_0 \cdot sin\,\theta \cdot t_y \cdot sin\,\varphi \cdot \cos(\omega t - \delta)] \cdot \mathbf{n}, \tag{6}$$

Where φ is the angle between the direction of analyzer and the fast axis. To simplify the formula, we define $A = E_0 \cdot cos\,\theta \cdot t_x$ and $B = E_0 \cdot sin\,\theta \cdot t_y$. Accordingly, by taking the time average within a cycle to eliminate t, we can obtain the expression for transmitted intensity I and angle φ:

$$I(\varphi) = \frac{1}{T}\int_0^T |A \cdot cos\,\varphi \cdot \cos(\omega t) + B \cdot sin\,\varphi \cdot \cos(\omega t - \delta)|^2 dt \qquad (\omega T = 2\pi)$$

$$= \frac{1}{2}(A^2 \cdot cos^2\,\varphi + B^2 \cdot sin^2\,\varphi + 2AB \cdot sin\,\varphi \cdot cos\varphi \cdot cos\delta). \tag{7}$$

Polarization-dependent intensity of input/output spectra were well fitted by Supplementary Equation 7 in the main article. By further simplification, we can get the following expression:

$$I(\varphi) = \frac{A^2+B^2}{4} + \frac{1}{2}\sqrt{\left(\frac{A^2-B^2}{2}\right)^2 + (ABcos\delta)^2} \cdot \sin\left(2\varphi + \arctan\left(\frac{A^2-B^2}{2ABcos\delta}\right)\right) \tag{8}$$

Thus, I_{max} and I_{min} can be obtained as follow:

$$I_{max} = \frac{A^2+B^2}{4} + \frac{1}{2}\sqrt{\left(\frac{A^2-B^2}{2}\right)^2 + (ABcos\delta)^2}, \tag{9}$$

$$I_{min} = \frac{A^2+B^2}{4} - \frac{1}{2}\sqrt{\left(\frac{A^2-B^2}{2}\right)^2 + (ABcos\delta)^2}. \quad (10)$$

Hence, we can get the expression of DOP:

$$DOP = \frac{I_{max}-I_{min}}{I_{max}+I_{min}} = \frac{2\sqrt{\left(\frac{A^2-B^2}{2}\right)^2+(ABcos\delta)^2}}{A^2+B^2}. \quad (11)$$

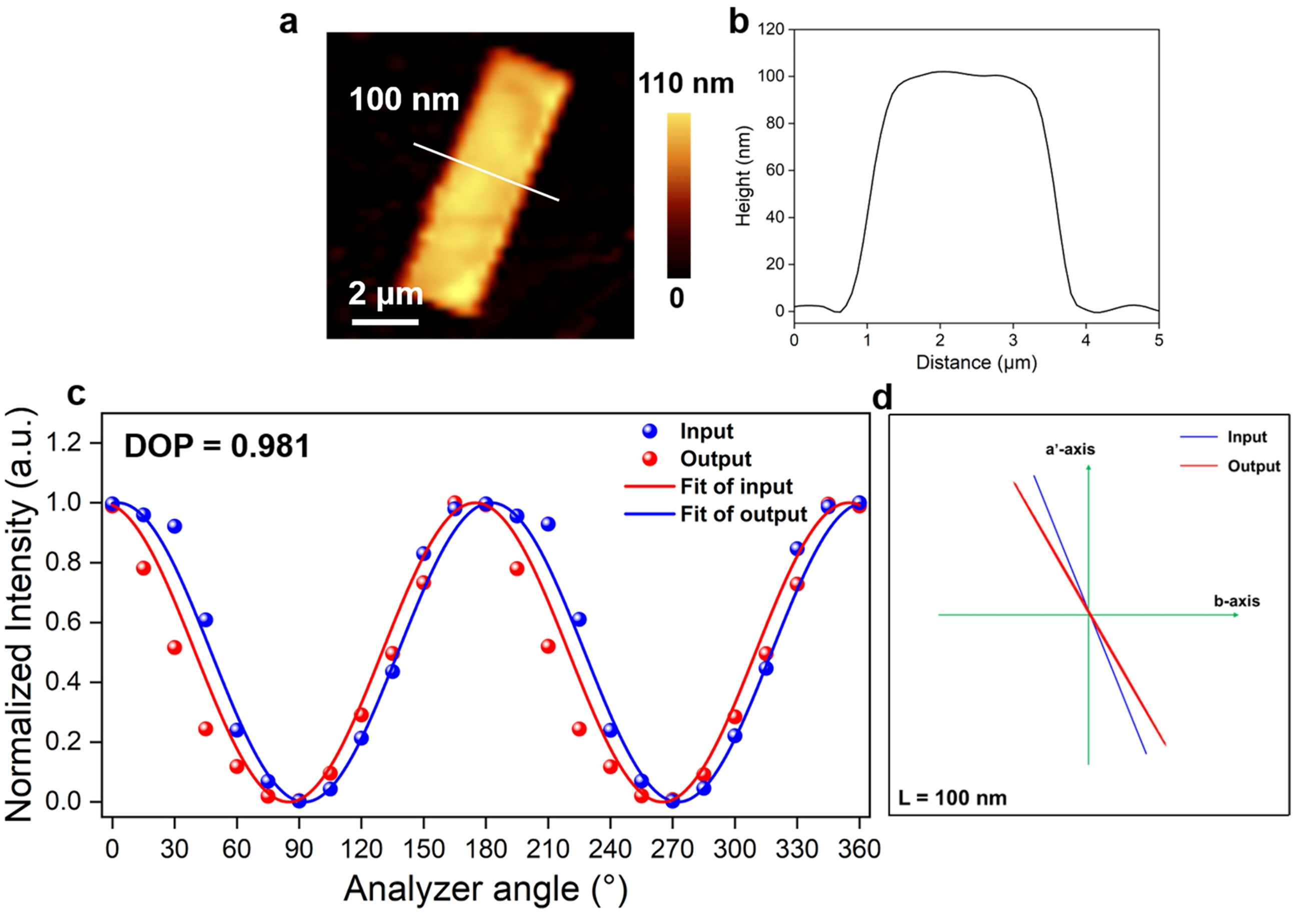

Supplementary Fig. 41 a AFM images, and **b** the corresponding line profiles of the fibrous RP flakes with a thickness of 100 nm. **c** Polarization-dependent intensity of input/output spectra for the fibrous RP flake corresponding to Supplementary Fig. 41a. **d** Schematic diagram of polarization state for input/output light corresponding to Supplementary Fig. 41c.

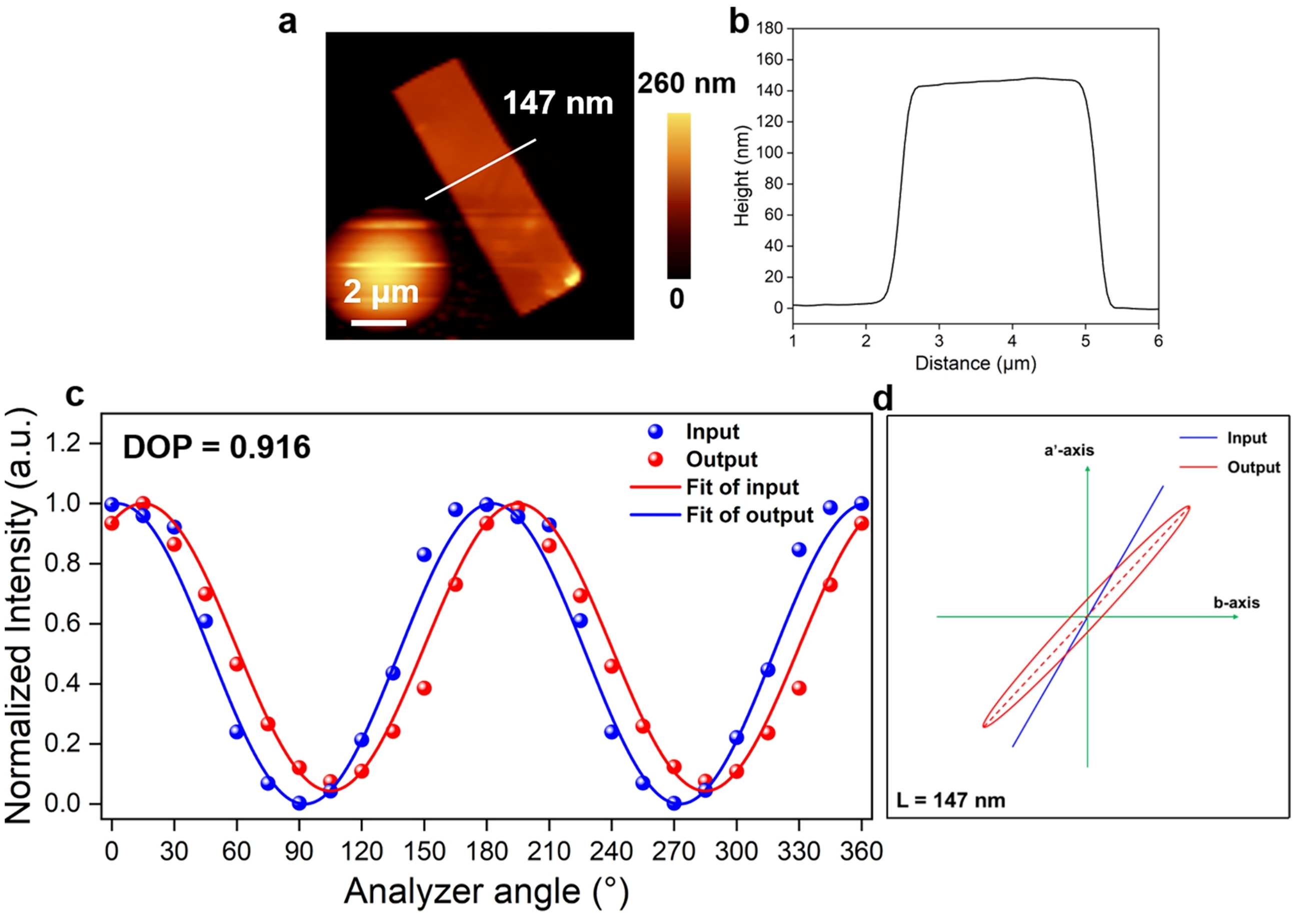

Supplementary Fig. 42 **a** AFM images, and **b** the corresponding line profiles of the fibrous RP flakes with a thickness of 147 nm. **c** Polarization-dependent intensity of input/output spectra for the fibrous RP flake corresponding to Supplementary Fig. 42a. **d** Schematic diagram of polarization state for input/output light corresponding to Supplementary Fig. 42c.

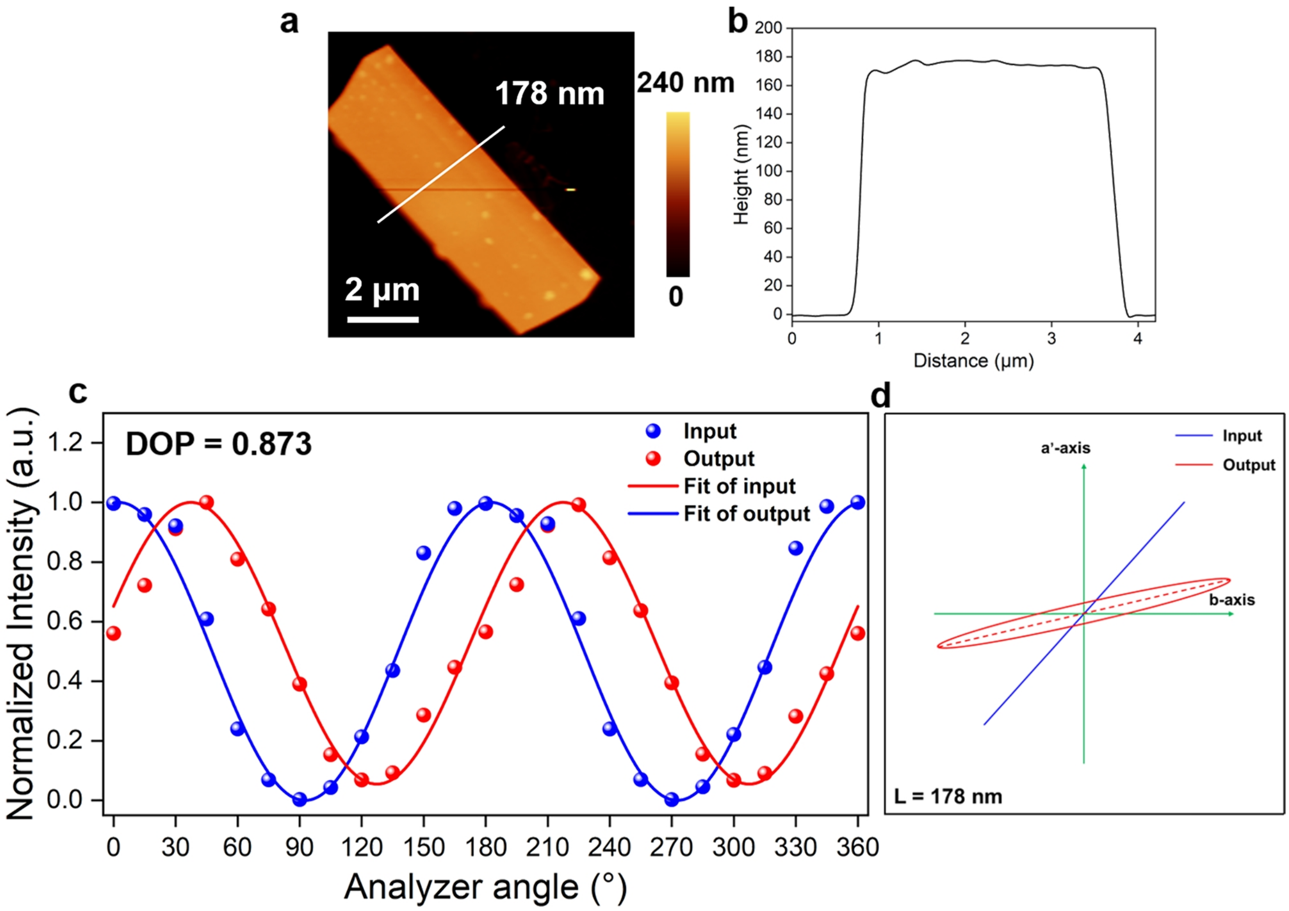

Supplementary Fig. 43 a AFM images, and **b** the corresponding line profiles of the fibrous RP flakes with a thickness of 178 nm. **c** Polarization-dependent intensity of input/output spectra for the fibrous RP flake corresponding to Supplementary Fig. 43a. **d** Schematic diagram of polarization state for input/output light corresponding to Supplementary Fig. 43c.

The phase retardation value δ can be derived using Supplementary Equation 12:

$$cos^2\delta = \frac{DOP^2(A^2+B^2)^2-(A^2-B^2)^2}{4A^2B^2}. \quad (12)$$

Combining $\delta = \Delta n 2\pi d/\lambda$, we can further obtain the in-plane refractive index difference at 532 nm. The extracted constants are exhibited in the following Supplementary Table 5:

Supplementary Table 5 The correlation constants obtained by extraction.

$d\ (nm)$	DOP	δ	$\Delta n'$	$\Delta\bar{n}'$	$\Delta n'_{cal}$
64	0.995	0.185792	0.245798		
100	0.981	0.28811	0.243944		
147	0.916	0.488021	0.281095	0.26171±0.02359	0.513
178	0.873	0.514115	0.244552		
596	0.8	2.063419	0.293139		

Supplementary References

1. Liu, X, Zhang, X., Lin, M. and Tan, P. Different angle-resolved polarization configurations of Raman spectroscopy: A case on the basal and edge plane of two-dimensional materials. *Chin. Phys. B* **26**, 067802 (2017).
2. Feng, X. *et al*. 2D Inorganic Bimolecular Crystals with Strong In-Plane Anisotropy for Second-Order Nonlinear Optics. *Adv. Mater.* **32**, 2003146 (2020).
3. Zhao, H. *et al*. Interlayer interactions in anisotropic atomically thin rhenium diselenide. *Nano Res.* **8**, 3651-3661 (2015).
